# Supplementary material for: Maternal Obesity and Patterns in Postnatal Diet, Physical Activity and Weight among a Highly Deprived Population in the UK: The GLOWING Pilot Trial
Source: Nutrients. 2023 Aug 30;15(17):3805. doi: 10.3390/nu15173805 (PMC10490453; doi:10.3390/nu15173805)
Supplement: Supplementary file 1 [file nutrients-15-03805-s001.zip › nutrients-2550750-supplementary.pdf]

Supplementary Material

Table S1: Comparison of dietary patterns between intervention and control groups at 3-, 6-, 9- and 12-months postnatal.

	**Median (IQR) intake at each postnatal time point**											
	**3-months**			**6-months**			**9-months**			**12-months**		
	Intervention (n=14)	*Control (n=10)*	*P-Value*	*Intervention (n=12)*	*Control (n=10)*	*P-Value*	*Intervention (n=4)*	*Control (n=8)*	*P-Value*	*Intervention (n=13)*	*Control (n=7)*	*P-Value*
Milk ml/day	142 (107, 285)	214 (142, 285)	0.172	142 (142, 285)	214 (142, 320)	0.530	214 (142, 285)	142 (142, 285)	0.571	142 (142, 285)	142 (0, 285)	0.709
Spread g/day	6 (2, 10)	6 (0, 17)	0.791	8 (3, 18)	10 (4, 27)	0.593	13 (7, 19)	9 (3, 11)	0.305	6 (3, 10)	3 (0, 6)	0.141
Cheese g/day	2 (0, 11)	23 (0, 42)	0.071	8 (1, 33)	13 (0, 47)	0.790	1 (0, 30)	5 (1, 22)	0.489	9 (2, 20)	15 (0, 51)	0.841
Sugary drinks ml/day	150 (64, 273)	200 (20, 509)	0.776	209 (28, 521)	14 (0, 80)	0.029	127 (0, 1014)	14 (0, 80)	0.724	240 (17, 724)	14 (0, 402)	0.175
Fruit Juice	0 (0, 28)	0 (0, 28)	0.974	0 (0, 85)	7 (0, 17)	0.638	7 (0, 24)	7 (0, 24)	0.927	14 (0, 28)	14 (0, 14)	0.736
Sugar-sweetened beverages	139 (0, 263)	200 (0, 495)	0.749	200 (28, 521)	0 (0, 73)	0.022	113 (0, 997)	0 (0, 28)	0.312	86 (0, 501)	0 (0, 402)	0.219
Starchy carbohydrates g/day	94 (61, 143)	103 (54, 141)	1.000	85 (46, 124)	152 (81, 180)	0.044	135 (88, 192)	109 (77, 146)	0.450	91 (42, 134)	55 (24, 161)	0.500
Rice, pasta, noodles, potatoes	14 (12, 17)	15 (12, 17)	0.849	15 (12, 17)	16 (14, 20)	0.401	15 (11, 19)	16 (11, 20)	0.865	15 (13, 19)	16 (12, 21)	0.551
Takeaway & oven chips	4 (3, 5)	5 (4, 6)	0.112	5 (3, 6)	5 (4, 6)	0.632	8 (5, 10)	5 (2, 6)	0.038	4 (4, 6)	6 (4, 6)	0.487
Bread	46 (24, 83)	72 (27, 90)	0.874	39 (21, 72)	51 (46, 108)	0.124	108 (71, 144)	72 (51, 108)	0.217	41 (25, 108)	31 (0, 51)	0.299
Breakfast cereal	11 (0, 34)	10 (0, 26)	0.903	0 (0, 29)	17 (0, 91)	0.103	4 (0, 20)	0 (0, 29)	0.686	0 (0, 24)	0 (0, 38)	0.963
Fruit and vegetables g/day	223 (75, 448)	95 (64, 191)	0.186	150 (58, 317)	147 (63, 256)	0.939	129 (54, 131)	221 (41, 360)	0.308	176 (65, 302)	102 (21, 123)	0.191
Vegetables	125 (57, 191)	64 (29, 113)	0.188	86 (50, 192)	66 (29, 94)	0.355	63 (34, 87)	75 (20, 136)	0.932	87 (24, 132)	60 (9, 102)	0.303
Fruits	69 (18, 282)	41 (17, 101)	0.468	55 (8, 139)	102 (25, 163)	0.394	47 (10, 74)	110 (36, 176)	0.202	53 (24, 195)	29 (12, 57)	0.218
Snacks g/day	34 (20, 66)	74 (32, 128)	0.102	51 (21, 86)	70 (24, 165)	0.522	181 (67, 208)	45 (13, 64)	0.042	45 (14, 79)	25 (14, 34)	0.322
Crisps and fried snacks	2 (2, 4)	3 (2, 5)	0.437	5 (3, 5)	5 (4, 5)	0.700	4 (2, 8)	4 (2, 5)	0.727	2 (2, 4)	3 (2, 5)	0.541
Sweet snacks	27 (18, 55)	53 (28, 109)	0.089	45 (15, 80)	61 (16, 157)	0.570	174 (60, 201)	4 (8, 56)	0.062	40 (10, 73)	21 (5, 27)	0.234
Yoghurt (g/day)	2 (1, 5)	3 (2, 4)	0.950	2 (1, 4)	4 (2, 4)	0.105	2.00 (1.25, 5.00)	4 (1, 6)	0.793	3 (1, 4)	2 (1, 4)	0.560
Meat and fish g/day	139 (83, 233)	162 (122, 216)	0.639	185 (137, 228)	158 (116, 189)	0.414	209 (106, 479)	145 (92, 174)	0.308	132 (89, 173)	90 (56, 172)	0.452

	Median (IQR) intake at each postnatal time point											
	**3-months**			**6-months**			**9-months**			**12-months**		
	Intervention (n=14)	*Control (n=10)*	*P-Value*	*Intervention (n=12)*	*Control (n=10)*	*P-Value*	*Intervention (n=4)*	*Control (n=8)*	*P-Value*	*Intervention (n=13)*	*Control (n=7)*	*P-Value*
Red meat	22 (11, 82)	68 (8, 68)	0.788	68 (22, 125)	68 (17, 68)	0.511	96 (25, 150)	40 (0, 68)	0.091	22 (22, 68)	22 (11, 68)	0.533
White meat	57 (88)	57 (57, 111)	0.308	57 (28, 92)	57 (57, 57)	0.908	57 (21, 113)	57 (21, 57)	0.792	18 (18, 81)	18 (9, 57)	0.653
Processed meat	15 (7, 59)	19 (10, 19)	0.976	19 (12, 59)	19 (10, 19)	0.216	39 (19, 118)	15 (2, 19)	0.029	19 (10, 39)	10 (0, 19)	0.232
Processed fish	9 (0, 9)	9 (0, 18)	0.639	0 (0, 9)	9 (0, 14)	0.242	9 (0, 82)	5 (0, 16)	0.583	9 (0, 14)	9 (0, 18)	0.966
Oily Fish	0 (0, 11)	0 (0, 11)	0.672	0 (0, 7)	0 (0, 35)	0.475	9 (2, 42)	0 (0, 17)	0.466	0 (0, 4)	0 (0, 17)	0.186
White fish	7 (0, 14)	7 (5, 9)	0.709	0 (0, 7)	14 (7, 29)	0.005	4 (0, 7.)	11 (7, 14)	0.028	7 (0, 11)	7 (0, 14)	0.832

All data presented as median (IQR) due to inconsistency in normality of distribution using Shapiro-Wilkes test. Statistical significance $p<0.05$, p-value derived from Mann-Whitney U test comparing dietary intake between intervention and control groups.

Table S2: Comparison of physical activity behaviours between intervention and control groups at 3-, 6-, 9- and 12-months postnatal.

	Median (IQR) energy expenditure at each postnatal time point											
	**3-months**			**6-months**			**9-months**			**12-months**		
	Intervention (n=14)	*Control* (n=10)	*P-value*	*Intervention* (n=12)	*Control* (n=10)	*P-value*	*Intervention* (n=4)	*Control* (n=8)	*P-value*	*Intervention* (n=13)	*Control* (n=7)	*P-value*
Total Energy Expenditure	264.73 (155.47, 315.96)	188.05 (120.22, 265.05)	0.19	245.93 (178.79, 361.33)	202.08 (131.38, 269.20)	0.20	155.07 (118.23, 317.49)	249.08 (215.52, 313.17)	0.23	385.68 (224.68, 467.74)	214.92 (162.25, 306.10)	0.14
Sedentary PA	17.85 (13.03, 30.01)	17.85 (13.03, 32.90)	0.89	12.60 (4.42, 26.78)	17.85 (14.92, 17.85)	0.63	17.85 (9.98, 26.51)	17.85 (17.85, 29.40)	0.53	17.85 (7.35, 23.39)	17.85 (7.35, 30.80)	0.64
Light PA	142.73 (82.43, 155.53)	89.44 (69.60, 115.60)	0.11	131.06 (98.16, 158.87)	98.79 (66.12, 152.70)	0.38	101.66 (64.45, 204.31)	111.69 (102.20, 146.01)	0.65	186.90 (120.42, 213.81)	110.56 (67.87, 134.19)	0.02
Moderate PA	96.13 (69.20, 159.53)	78.86 (27.02, 111.27)	0.37	114.03 (63.16, 147.40)	57.11 (38.50, 101.33)	0.07	35.57 (24.96, 105.52)	83.57 (76.32, 159.25)	0.11	166.83 (65.01, 252.80)	91.79 (66.36, 122.74)	0.39
Vigorous PA	0.39 (0.00, 0.78)	0.78 (0.00-3.31)	0.47	0.00 (0.00, 2.68)	0.39 (0.00, 0.78)	0.87	0.00 (0.00, 0.00)	0.00 (0.00, 1.62)	0.53	0.00 (0.00, 6.89)	0.00 (0.00, 0.78)	0.64
Household/ care PA	170.84 (109.13, 262.38)	155.01 (81.85, 180.76)	0.24	186.74 (133.47, 275.81)	120.20 (82.49, 206.01)	0.08	115.46 (71.91, 129.46)	164.39 (101.22, 180.60)	0.16	213.85 (83.72, 262.54)	131.53 (123.03, 226.20)	1.00
Occupational PA	0.00 (0.00, 0.00)	0.00 (0.00, 0.00)	1.00	0.00 (0.00, 0.00)	0.00 (0.00, 0.00)	0.46	0.00 (0.00, 0.00)	0.00 (0.00, 88.87)	0.79	71.57 (0.00, 182.00)	0.00 (0.00, 71.82)	0.21
Sport PA	7.16 (1.43, 12.58)	4.42 (1.71, 17.76)	0.75	3.73 (0.10, 7.82)	3.98 (1.60, 5.23)	0.82	0.19 (0.00, 6.62)	1.60 (0.38, 5.25)	0.32	1.60 (0.00, 18.61)	1.60 (0.38, 4.68)	0.88
Transport PA	29.68 (19.37, 58.94)	11.41 (8.66, 38.06)	0.14	27.23 (20.44, 37.70)	10.71 (9.19, 23.96)	0.02	12.04 (10.19, 28.54)	21.35 (12.11, 22.61)	0.53	28.00 (12.04, 59.50)	14.00 (1.26, 17.36)	0.04
Inactive PA	18.38 (15.23, 33.36)	24.15 (13.03, 34.03)	0.89	12.60 (4.80, 33.34)	17.85 (17.12, 21.96)	0.31	24.91 (10.35, 34.39)	29.40 (17.85, 30.45)	1.00	17.85 (7.35, 43.04)	17.85 (8.86, 35.70)	0.76

All data presented as median (IQR) due to inconsistency in normality of distribution using Shapiro-Wilkes test. Statistical significance $p<0.05$, p-value derived from Mann-Whitney U test comparing physical activity between intervention and control groups.

Table S3: Comparison of weight change between intervention and control groups at 3-, 6-, 9- and 12-months postnatal

	**Median (IQR) weight change at each postnatal time point**											
	**3-months**			**6-months**			**9-months**			**12-months**		
	Intervention (n=12)	*Control* (n=8)	*P-value*	*Intervention* (n=12)	*Control* (n=10)	*P-value*	*Intervention* (n=4)	*Control* (n=6)	*P-value*	*Intervention* (n=13)	*Control* (n=7)	*P-value*
Weight change from booking	0.1 (-7.9, 4.1)	-6.0 (-9.7, -2.2)	0.123	0.9 (-7.0, 4.7)	-3.0 (-5.9, 4.9)	0.792	4.4 (-0.7, 8.4)	-2.0 (-3.6, -0.3)	0.088	2.0 (-9.3, 4.3)	-2.3 (-5.5, -0.3)	0.452
	Intervention (n=10)	*Control* (n=8)	*P-value*	*Intervention* (n=7)	*Control* (n=9)	*P-value*	*Intervention* (n=1)	*Control* (n=6)	*P-value*	*Intervention* (n=10)	*Control* (n=5)	*P-value*
Weight change from 3rd trimester	-11.4 (-15.4, -4.9)	-11.6 (-14.8, -9.2)	0.722	-9.8 (-13.8, -4.9)	-11.8 (-12.4, -5.5)	0.958	8.0	-9.8 (-11.2,-6.1)	0.134	-6.8 (-10.8, -0.9)	-9.0 (-10.6, -7.4)	0.540

All data presented as median (IQR) due to inconsistency in normality of distribution using Shapiro-Wilkes test. Statistical significance p<0.05, p-value derived from Mann-Whitney U test comparing weight change between intervention and control groups.

Table S4: STROBE reporting guidelines checklist

	Item No	Recommendation	Page No
**Title and abstract**	1	(*a*) Indicate the study's design with a commonly used term in the title or the abstract	1
		(*b*) Provide in the abstract an informative and balanced summary of what was done and what was found	1
**Introduction**			
Background/rationale	2	Explain the scientific background and rationale for the investigation being reported	1-2
Objectives	3	State specific objectives, including any prespecified hypotheses	2
**Methods**			
Study design	4	Present key elements of study design early in the paper	2-3
Setting	5	Describe the setting, locations, and relevant dates, including periods of recruitment, exposure, follow-up, and data collection	3
Participants	6	(*a*) Give the eligibility criteria, and the sources and methods of selection of participants. Describe methods of follow-up<br>(*b*) For matched studies, give matching criteria and number of exposed and unexposed	3
Variables	7	Clearly define all outcomes, exposures, predictors, potential confounders, and effect modifiers. Give diagnostic criteria, if applicable	3-4
Data sources/ measurement	8*	For each variable of interest, give sources of data and details of methods of assessment (measurement). Describe comparability of assessment methods if there is more than one group	*3-4*
Bias	9	Describe any efforts to address potential sources of bias	4
Study size	10	Explain how the study size was arrived at	3
Quantitative variables	11	Explain how quantitative variables were handled in the analyses. If applicable, describe which groupings were chosen and why	4
Statistical methods	12	(*a*) Describe all statistical methods, including those used to control for confounding<br>(*b*) Describe any methods used to examine subgroups and interactions<br>(*c*) Explain how missing data were addressed<br>(*d*) If applicable, explain how loss to follow-up was addressed<br>(*e*) Describe any sensitivity analyses	2-4
**Results**			
Participants	13*	(a) Report numbers of individuals at each stage of study—eg numbers potentially eligible, examined for eligibility, confirmed eligible, included in the study, completing follow-up, and analysed<br>(b) Give reasons for non-participation at each stage<br>(c) Consider use of a flow diagram	4

Descriptive data	14*	(a) Give characteristics of study participants (eg demographic, clinical, social) and information on exposures and potential confounders<br>(b) Indicate number of participants with missing data for each variable of interest<br>(c) Summarise follow-up time (eg, average and total amount)	4
Outcome data	15*	Report numbers of outcome events or summary measures over time	5-7
Main results	16	(*a*) Give unadjusted estimates and, if applicable, confounder-adjusted estimates and their precision (eg, 95% confidence interval). Make clear which confounders were adjusted for and why they were included	n/a
		(*b*) Report category boundaries when continuous variables were categorized	n/a
		(*c*) If relevant, consider translating estimates of relative risk into absolute risk for a meaningful time period	n/a
Other analyses	17	Report other analyses done—eg analyses of subgroups and interactions, and sensitivity analyses	n/a
**Discussion**			
Key results	18	Summarise key results with reference to study objectives	8
Limitations	19	Discuss limitations of the study, taking into account sources of potential bias or imprecision. Discuss both direction and magnitude of any potential bias	10
Interpretation	20	Give a cautious overall interpretation of results considering objectives, limitations, multiplicity of analyses, results from similar studies, and other relevant evidence	8-10
Generalisability	21	Discuss the generalisability (external validity) of the study results	10
**Other information**			
Funding	22	Give the source of funding and the role of the funders for the present study and, if applicable, for the original study on which the present article is based	11

Table S5: Socio-demographic characteristics of women at 36-weeks' gestation and 3-, 6-, 9- and 12-months postnatal compared to women at 12-weeks' gestation.

	**36-wk**			**3-months**			**6-months**			**9-months**			**12-months**		
	**Missing** (n=65)	**Returned** (n=39)	**P-value**	**Missing** (n=80)	**Returned** (n=24)	**P-value**	**Missing** (n=82)	**Returned** (n=22)	**P-value**	**Missing** (n=92)	**Returned** (n=12)	**P-value**	**Missing** (n=84)	**Returned** (n=20)	**P-value**
Booking BMI, kg/m² (median, IQR)	35.0 (32.9, 39.0)	34.4 (32.5, 41.6)	0.660	34.9 (32.7, 39.0)	35.0 (32.9, 42.8)	0.594	34.6 (32.3, 41.6)	35.9 (33.8, 39.0)	0.360	34.8 (32.7, 41.7)	36.6 (33.9, 39.0)	0.597	34.8 (32.4, 39.0)	36.0 (33.2, 45.2)	0.220
Maternal age, years (mean, SD)	29.3 (5.0)	30.8 (5.9)	0.177	29.5 (5.6)	31.0 (4.27)	0.231	31.2 (4.6)	29.5 (5.5)	0.203	29.9 (5.3)	30.1 (5.9)	0.894	30.1 (5.4)	29.0 (5.4)	0.413
Number of pregnancies (median, IQR)	2 (2, 4)	2 (1, 4)	0.608	2 (2, 4)	2 (1, 3)	0.362	2 (2, 4)	2 (1, 4)	0.507	2 (1, 4)	2 (2, 4)	0.949	2 (2, 2)	2 (1, 4)	0.421
Number of pregnancies >24 weeks (Median, IQR)	2 (0, 2)	2 (0, 2)	0.131	2 (0, 2)	2 (0, 2)	0.344	2 (0, 2)	2 (0, 2)	0.780	2 (0, 2)	2 (0, 2)	0.621	2 (0, 2)	2 (0, 2)	0.194
Deprivation quintile, n (%)															
Quintile 1&2	49 (76.6)	25 (65.8)	0.302	58 (73.4)	16 (69.6)	0.851	58 (71.6)	16 (76.2)	0.784	64 (71.1)	10 (83.3)	na	58 (70.7)	16 (80)	0.390
Quintile 3-5	15 (23.4)	13 (34.2)		21 (26.6)	7 (30.4)		23 (28.4)	5 (23.8)		26 (28.9)	2 (16.7)		24 (29.3)	4 (20)	
Usual Employment, n (%)															
Employed	36 (57.1)	30 (76.9)	0.042	47 (60.3)	19 (79.2)	0.090	48 (60)	18 (81.8)	0.580	57 (63.3)	9 (75)	na	52 (63.4)	14 (70)	0.581
Unemployed	27 (42.9)	9 (23.1)		31 (39.7)	5 (20.8)		32 (40)	4 (18.2)		33 (36.7)	3 (25)		30 (36.6)	6 (30)	
Ethnic Group, n (%)															
White	62 (95.4)	37 (94.9)	na	77 (96.3)	22 (91.7)	na	79 (96.3)	20 (90.9)	na	89 (96.7)	10 (83.3)	na	80 (95.2)	19 (95)	na
Other	3 (4.6)	2 (5.1)		3 (3.8)	2 (8.3)		3 (3.7)	2 (9.1)		3 (3.3)	2 (16.7)		4 (4.8)	1 (5)	
Education, n (%)															
Education High School/GCSE or lower	34 (52.3)	16 (42.1)	0.317	37 (46.3)	13 (56.5)	0.385	42 (51.2)	8 (38.1)	0.283	46 (50.5)	4 (33.3)	0.262	42 (50.6)	8 (40)	0.394
Education High School/GCSE or higher	31 (47.7)	22 (57.9)		43 (53.8)	10 (43.5)		40 (48.8)	13 (61.9)		45 (49.5)	8 (66.7)		41 (49.4)	12 (60)	

	36-wk			3-months			6-months			9-months			12-months		
	**Missing** (n=65)	**Returned** (n=39)	**P-value**	**Missing** (n=80)	**Returned** (n=24)	**P-value**	**Missing** (n=82)	**Returned** (n=22)	**P-value**	**Missing** (n=92)	**Returned** (n=12)	**P-value**	**Missing** (n=84)	**Returned** (n=20)	**P-value**
Relationship status, n (%)															
Married	27 (41.5)	22 (57.9)	0.109	37 (46.3)	12 (52.2)	0.616	35 (43.2)	14 (63.6)	0.089	42 (46.2)	7 (58.3)	0.427	38 (45.8)	11 (55)	0.459
Not Married	38 (58.5)	16 (42.1)		43 (53.8)	11 (47.8)		46 (56.8)	8 (36.4)		49 (53.8)	5 (41.7)		45 (54.2)	9 (45)	

Categories collapsed to increase sample size in some variables. Normally distributed data presented as mean (SD). Non-normally distributed data presented as median (IQR). Statistical significance $p<0.05$, p-value derived from Mann-Whitney U test, t-test or chi-squared test comparing socio-demographics for missing and returned questionnaires. Chi-squared test possible in some due to expected values $n \geq 5$, however Chi-squared test not possible in some due to expected values $n<5$.

Table S6: Participant socio-demographic characteristics at 3-, 6-, 9 -and 12-months postnatal.

		3-months (n=24)	6-months (n=22)	9-months (n=12)	12-months (n=20)
Booking BMI, kg/m²	Median (IQR)	35.0 (32.9, 42.8)	35.9 (33.8, 39.0)	36.6 (33.9, 39.0)	36.0 (33.2, 45.2)
	Class 1 n (%)	12 (50.0)	9 (40.9)	5 (41.7)	9 (45.0)
	Class 2 n (%)	4 (16.7)	9 (40.9)	6 (50.0)	5 (25.0)
	Class 3 n (%)	8 (33.3)	4 (18.2)	1 (8.3)	6 (30.0)
Maternal age, years	Mean (SD)	31.0 (4.3)	31.2 (4.6)	30.1 (5.9)	29.0 (5.4)
Number of pregnancies	Median (IQR)	2 (1, 3)	2 (1, 3)	2 (2, 4)	2 (1, 4)
Number of pregnancies progressing >24 weeks	Median (IQR)	2 (1, 3)	2 (1, 3)	2 (1, 3)	2 (1, 2)
Deprivation quintile, n (%)	Q1 (most deprived)	11 (45.8)	12 (54.5)	7 (58.3)	13 (65.0)
	Q2	5 (20.8)	4 (18.2)	3 (25.0)	3 (15.0)
	Q3	2 (8.3)	1 (4.5)	0	1 (5.0)
	Q4	4 (16.7)	4 (18.2)	2 (16.7)	3 (15.0)
	Q5 (least deprived)	1 (4.2)	0	0	0
	Missing	1 (4.2)	1 (4.5)	0	0
Usual Employment, n (%)	Employed full time	15 (62.5)	13 (59.1)	6 (50.0)	11 (55.0)
	Employed part time	4 (16.7)	3 (13.6)	2 (16.7)	3 (15.0)
	Self-employed	0	2 (9.1)	1 (8.3)	0
	Unemployed	4 (16.7)	3 (13.6)	3 (25.0)	5 (25.0)
	Full-time student	0	0	0	0
	Unpaid carer for family/friend	1 (4.2)	1 (4.5)	0	0
	Other [a]	0	0	0	1 (5.0)
Ethnic Group, n (%)[b]	White	22 (91.7)	20 (90.9)	10 (83.3)	19 (95.0)
	South Asian	1 (4.2)	1 (4.5)	0	0
	Mixed ethnic group	1 (4.2)	1 (4.5)	1 (8.3)	1 (5.0)
	Other ethnic group	0	0	1 (8.3)	0
Education, n (%)	No formal qualifications	2 (8.3)	0	0	2 (10.0)
	GCSEs or equivalent	11 (45.8)	8 (36.3)	4 (33.3)	6 (30.0)
	A-Levels or equivalent	7 (21.2)	7 (31.8)	2 (16.7)	7 (35.0)
	Bachelor's degree or higher	2 (8.3)	3 (13.6)	3 (25.0)	4 (20.0)
	Other [c]	1 (4.2)	3 (13.6)	3 (25.0)	1 (5.0)
	Missing	1 (4.2)	1 (4.5)	0	0

		3-months (n=24)	6-months (n=22)	9-months (n=12)	12-months (n=20)
Relationship status, n (%)	Single	11 (45.8)	7 (31.8)	5 (41.7)	9 (45.0)
	Married	12 (50.0)	14 (63.6)	7 (58.3)	11 (55.0)
	Separated/divorced/widowed	0	1 (4.5)	0	0
	Missing	1 (4.2)	0	0	0
Smoking Status, n (%)	Never smoked	13 (54.2)	14 (63.6)	7 (58.3)	11 (55.0)
	Smoked in the past but not during pregnancy	6 (25.0)	5 (22.7)	0	0
	Not smoking now (smoked in pregnancy and not using NRT)	0	0	0	3 (15.0)
	Not smoking now (smoked in pregnancy and using NRT)	0	0	2 (16.7)	2 (10.0)
	Currently smoke	5 (20.8)	3 (13.6)	3 (25.0)	4 (20.0)
	If currently smoke approx. n of cigarettes/day (mean, SD)	7.8 (4.8)	12.0 (8.0)	10 (0.0)	12.5 (8.7)
Currently breastfeeding? n (%)	Exclusively breastfeeding	3 (12.5)	4 (18.2)	1 (8.3)	0
	Breastfeeding + formula	2 (8.3)	2 (9.1)	0	0
	Exclusively breastfed for a while but no longer	2 (8.3)	4 (18.2)	3 (25.0)	3 (15.0)
	Breastfed + formula fed but no longer breastfeeding	2 (8.3)	2 (9.1)	0	7 (35.0)
	Never breastfed	15 (62.6)	10 (45.5)	8 (66.7)	10 (50.0)
Dietary Preference, n (%)	Both meat and fish	17 (70.8)	16 (72.7)	9 (75.0)	17 (85.0)
	Avoid meat but eat fish	2 (8.3)	1 (4.5)	0	0
	Avoid fish but eat meat	5 (20.8)	5 (22.7)	3 (25.0)	3 (15.0)
	Vegetarian	0	0	0	0
Do you currently drink alcohol? n (%)	No	12 (50.0)	13 (59.1)	3 (25.0)	13 (65.0)
	Yes	12 (50.0)	9 (40.9)	9 (75.0)	7 (35.0)
	If yes, approximate number of units/week *(median, IQR)*	3 (2, 4)	2 (2, 4.75)	2 (1.5, 4)	3 (2, 6)

The categories include: (a) Other employment: housewife, paid carer, zero-hours contact and traineeship; (b) Ethnic group: White (White British, White Irish, Other White), Black (Black Caribbean, Black African, Other Black), South Asian (Bangladeshi, Indian, Pakistani, Other South Asian, mixed ethnic group (White and Asian, White and Black African, White and Black Caribbean, Other Mixed), other ethnic groups (Chinese, Arabic, any other group) and prefer not to say; (c) Other education: Diploma, Apprenticeship, General National Vocational Qualification (GNVQ), and National Vocational Qualification (NVQ); (d) Relationship status: single (including never married/civil partnership), separated (still legally married/civil partnership), married (including civil partnership), divorced (including legally dissolved civil partnership) and widowed (including civil partnership). There was no relationship status option for women who were co-habiting, therefore they may have self-defined as any relationship category.
